# Embodied Irrationality? Knowledge Avoidance, Willful Ignorance, and the Paradox of Autonomy

**DOI:** 10.3389/fpsyg.2021.769591

**Published:** 2021-11-26

**Authors:** Selene Arfini, Lorenzo Magnani

**Affiliations:** Computational Philosophy Laboratory, Philosophy Section, Department of Humanities, University of Pavia, Pavia, Italy

**Keywords:** knowledge avoidance, willful ignorance, embodied cognition, epistemic feelings, self-deception, autonomy, hope, bounded rationality

## Abstract

In the current philosophical and psychological literature, *knowledge avoidance* and *willful ignorance* seem to be almost identical conditions involved in irrational patterns of reasoning. In this paper, we will argue that not only these two phenomena should be distinguished, but that they also fall into different parts of the epistemic rationality-irrationality spectrum. We will adopt an epistemological and embodied perspective to propose a definition for both terms. Then, we will maintain that, while willful ignorance is involved in irrational patterns of reasoning and beliefs, knowledge avoidance should be considered epistemically rational under particular circumstances. We will begin our analysis by considering which of the two phenomena is involved in patterns of reasoning that are still amply recognized as irrational—as wishful thinking, self-deception, and akrasia. We will then discuss the impact of epistemic feelings—which are emotional events that depend on epistemic states—on agents' decision-making. Then, we will consider the impact of willful ignorance and knowledge avoidance on agents' autonomy. By considering these issues, we will argue that when agents are aware that they are avoiding certain information (and aware of what kind of feelings acquiring the information would trigger), knowledge avoidance should be considered a rational, autonomy-increasing, hope-depended selection of information.

## Introduction

Various psychological studies have now confirmed that there are different situations in which the majority of people would not want to know something to avoid pain, regret, or anxiety (Eil and Rao, 2011; Sicherman et al., 2016; Gigerenzer and Garcia-Retamero, 2017). In some cases, people still choose to remain ignorant of something even if they would highly benefit, without apparent material costs, from the act of acquiring that information. For example, many patients who suffer from chronic diseases avoid getting information about their health even if having such knowledge is free and it would permit them to cope better, managing their symptoms and therapy (Oster et al., 2013). Still, a question that current literature strangely avoids is: is this cultivated ignorance epistemically irrational? For example, do these choices imply self-deception or do they affect agents' epistemic autonomy?^1^

Irrationality can be generally defined as a cognitive impediment (Bortolotti, 2010, 2014), and, more specifically, epistemic irrationality defines the creation of those beliefs which “are badly supported by the evidence available to the agent, or are maintained despite counter-evidence which is available to the agent” (Jefferson et al., 2017, p. 3). Since phenomena of *deliberate not-knowing* (terms that we will use to comprehend both willful ignorance and knowledge avoidance) involve the dismissal or the avoidance of evidence, it is reasonable to believe that there is a strong link between them and epistemic irrationality^2^. Contrary to this idea, in this paper, we argue that while willful ignorance can be rightfully considered as part of epistemically irrational patterns of reasoning, we can judge as epistemically rational the more specific condition of knowledge avoidance.

To advance our arguments, we will adopt an embodied cognition perspective. Thus, in section 1 we will comment on the fact that now discourses of rationality encompass, at various levels, takes from embodied cognition research and from theories of bounded/ecological rationality (Goldstein and Gigerenzer, 2002; Bissoto, 2007; Spellman and Schnall, 2009; Xu et al., 2020). Both these approaches have challenged the idea that irrationality involves only the deviance from rules of logic or probability. Also, emerging theories on ignorance have defied its definition as simply *lack of knowledge* or *true belief* , describing it as a more complex spectrum of states and processes (Arfini, 2019; Werner, 2021). Since now the distinctions between knowledge and ignorance and between rationality and irrationality are more blurred, we will argue that we need to consider *knowledge* avoidance different from willful *ignorance* and that they may fall into different parts of the rationality-irrationality spectrum. We will then propose a definition for both terms, grounded on the literature currently available.

Then, in section 2, we will consider that many irrational phenomena, such as wishful thinking (subsection 2.1), epistemic akrasia (subsection 2.2), and self-deception (subsection 2.3) require deliberate not-knowing. We will discuss which phenomenon between willful ignorance and knowledge avoidance is involved in these irrational processes, and we will argue that they mainly involve wishful ignorance but not knowledge avoidance (subsection 2.4).

In section 3, we will consider possible reasons to judge the phenomenon of knowledge avoidance as epistemically rational. Since the basic tenets of embodied cognition argue that bodily states affect cognitive processes (Chemero, 2011), we will argue that we should consider the emotional impact of certain information (in particular certain epistemic feelings, Arango-Muñoz, 2014a,b) among the costs of acquiring knowledge, contributing to labeling certain situations of knowledge avoidance as forms of rational ignorance. Then, in subsection 3.1, we will discuss the impact of knowledge avoidance on agents' autonomy, which will also bring us to discuss the paradox of autonomy, already introduced in Magnani (2020). By considering these issues and comparing cases of willful ignorance and knowledge avoidance (in subsection 3.2), we will argue that when agents are aware that they are avoiding certain information (and aware of what kind of feelings acquiring the information would trigger), knowledge avoidance should be considered a *rational, autonomy-increasing, hope-depending* selection of information.

## 1. Embodied Rationality and the Knowledge-Ignorance Spectrum

“The rational human is neither rational nor human.” With these words Spellman and Schnall (2009) begin their essay on how the rationality paradigm has evolved in the last few decades to encompass a more realistic account of the imperfect and limited rational individual.

Bounded rationality theories indeed explained why the majority of people in ordinary situations would not adhere to the rules imposed by logic and probability or would not maximize their utility (Mastrogiorgio and Petracca, 2016). The reason is not that irrationality is a natural human tendency, but that both internal (mental) and external (environmental) constraints limit our possibilities, making us more apt to look out for satisfying (*satisficing*, in Simon's lexicon Simon, 1997) options for decision-making instead of optimal ones. More than a few scholars (Gigerenzer and Goldstein, 1996; Spellman and Schnall, 2009; Xu et al., 2020) have written on the fall of the standard normative paradigm of rationality, and different currents emerged from its ashes (as, for example, theories of “ecological rationality” developed by Todd and Gigerenzer, 2007, 2012). So, yes, the rational human described with the old-fashioned paradigm of rationality could not be classified as *rational* in the same way as the bounded and ecologically rational human—also called *homo heuristicus* (Bardone, 2011)—we are now taking into consideration. But what about the *human* part of it?

Spellman and Schnall (2009) argue, and we agree, that those ideal cognizers who make decisions without considering their context nor their bodily cues have nothing of the human traits that characterize our typical agents. For this reason, bounded rationality today is variously rethought within the broader compass of embodied cognition research (Gallagher, 2018; Xu et al., 2020). Indeed, different principles of embodied cognition have poured into current theories of rationality and orient them into analyzing not only the individual cognizers but the cognitive system that comprehends them (Gallagher, 2018). Nonetheless, some patterns of reasoning, such as epistemic akrasia, self-deception, and wishful thinking, are still clearly epistemically irrational. They usually compromise instead of favoring good decision-making performances, and they do involve forms of deliberate not-knowing. Our question here is: which phenomena of deliberate not-knowing do these irrational conditions involve?

To answer this question, we should first provide reasons to consider willful ignorance and knowledge avoidance two different phenomena. To do that, we will rely on two main arguments: the difference between the current epistemological analysis of “knowledge” and “ignorance” and the specific different usage of “knowledge avoidance” and “willful ignorance” in philosophical, psychological, and cognitive literature.

The first argument relies on the complexity of the terms “knowledge” and “ignorance” in current epistemology. Knowledge, considered either with the traditional tripartite view that sees it as composed by *true and justified beliefs* (Gettier, 1963) or with more fallibilist accounts (Haack and Kolenda, 1977), is considered a more or less stable but peculiar phenomenon. On the contrary, ignorance has been recently depicted as a more nuanced and diffused condition since its concept encompasses not only the epistemic status of agents but also their attitudes toward it (Haas and Vogt, 2015). For example, we take for granted that ignorance is involved in cases in which agents do not know facts, but also when they do not realize they are not able to do something, or when they do not realize they have committed some errors doing a particular task, or if they have doubts about their competence, or if they do not know that they are competent in certain areas. These cases are, of course, very distinct and differently refer to first-order ignorance (subjects do not know *p*), second-order ignorance (subjects do not know whether they know *p*), or a mix of both, and in specialized literature they come with specific terminology, as *factual ignorance, procedural ignorance, doubt, uncertainty, error, tacit knowledge*, and so on^3^. Moreover, some cases involve both agents' ignorance and partial knowledge, as know-that or know-how. Still, we resist the attribution of knowledge, even partial knowledge, in these cases, while we have no problem in recognizing how the agent's beliefs system, reasoning, and behavior are affected by ignorance. The reason is that we hold a higher standard for the attribution of knowledge rather than ignorance, and so we tend to distinguish, for example, knowledge from mere belief, while we use a broad meaning for ignorance to generally speak of lack of knowledge, but also lack of awareness, comprehension, or confidence. This lower standard for the attribution of ignorance explains why definitions of ignorance as lack of knowledge (Le Morvan, 2013) or lack of true beliefs (Le Morvan and Peels, 2016) are now broadly challenged. Indeed, they seem to defy the common use of ignorance as a broader term, which refers to a combination of epistemic lack (lack of information, knowledge, competence, etc.) and specific attitudes of self-awareness (doubt, uncertainty, unawareness, etc.).

We also need to point out that in recent times, externalist approaches have also grounded emerging theories on ignorance, defying its definition as something that has to do with only higher cognitive functions of the individual. Different scholars are proposing embodied, extended, and distributed approaches to the idea of ignorance in both epistemological and psychological fields (Arfini, 2021; Arfini and Magnani, 2021; Werner, 2021). Thus, since there is an ampler spectrum of possibilities that defines what we call ignorance rather than what we recognize as knowledge, it is not unreasonable to argue that *knowledge avoidance* should be considered reasonably different from a state of *willful ignorance*.

In the usage of the two concepts, we can even see this difference. Knowledge or information avoidance is generally seen as the choice of not getting *specific information* for *particular reasons* (Sweeny et al., 2010). To make some examples, people may avoid acquiring certain knowledge:

- to postpone anxiety or pain regarding a specific situation (e.g., some patients avoid knowing if they have the genetic markers of a hereditary illness) (Sweeny et al., 2010; Eil and Rao, 2011);
- to preserve positive emotions, as awe and wonder, or even neutral ones, as surprise and suspense (e.g., some people avoid knowing the sex of the unborn child) (Gigerenzer and Garcia-Retamero, 2017).
- to preserve a fair judgment (e.g., the double-blind peer-review process) (Gigerenzer and Garcia-Retamero, 2017).

In all these cases, the agents avoid knowing a particular piece of information that may affect their judgment and reasoning. Instead, scholars often use “willful ignorance” to speak of the more general avoidance of situations that make someone aware of certain information, evidence, or knowledge. So, willful ignorance could prevent agents from knowing about the social impact of their decisions (Grossman and van der Weele, 2017), the law (Zimmerman, 2018), available information (Rubin, 2018), privileged perspectives (May, 2006), and make them disrespect the truth (McIntyre, 2015).

Various articles claim that the idea of “not wanting to know” must be a phenomenon so particular that it does not need any differentiation—which leads them to not distinguishing between willful ignorance and knowledge avoidance (Bertolotti et al., 2016; Gigerenzer and Garcia-Retamero, 2017). The problem with this kind of narrative is that it assumes that ignorance is more or less of one kind. However, intuitively and logically, we consider ignorance to be broader and more differentiated than knowledge. So it is not sufficient to say that ignorance is “what the agent is not aware of,” but also, for example, the kind of metacognitive judgments surrounding that ignorance.

Providing a functional definition, we can say that people are willfully ignorant of something when they avoid all circumstances that would allow them to acquire that knowledge, even by accident. Instead, people in a condition of knowledge avoidance do not perform the necessary steps to get a specific piece of information, which could not fall in their laps otherwise. As a last point of characterization, in the case of proper knowledge avoidance, the reasons for not wanting to know have nothing to do with the material costs of acquiring this knowledge, and the agent is also personally interested in acquiring this knowledge^4^.

Thus, the distinction between knowledge avoidance and willful ignorance should matter, given the new perspectives on rationality studies. Indeed, since the distinction between rationality and irrationality is now blurred, we need to argue that *knowledge avoidance* and *willful ignorance* may also fall into different parts of the rationality-irrationality spectrum. In the next section, we will then discuss which of the two phenomena is involved in some irrational patterns of reasoning, such as wishful thinking, self-deception, and epistemic akrasia. We will then maintain that most of these forms of irrationality involve wishful ignorance but not knowledge avoidance.

## 2. Discussion: Rational and Irrational Ignorance

First, we should point out a rule of thumb that may seem counter-intuitive prima facie but fairly simple to apply after a brief explanation: ignorance is not always epistemically bad for human agents, and knowledge is not always good either. In few words, we should be able to distinguish between a *rational* and *irrational ignorance*. Ignorance is usually presented as the rational choice when the costs of acquiring knowledge outweigh the benefits of possessing it (Mackie, 2012; Somin, 2015; Williams, 2021). In similar ways, also theories of bounded and ecological rationality suggest that we should consider knowledge a limited resource for some good reasons—even pragmatic ones (Jordan, 1996; Reisner, 2009; Star, 2018)^5^. If agents do not have enough time or computational capacity to get the appropriate data to make the most optimal choice, they need to rely on lesser goods.

Moreover, in this part of the analysis, we should consider the difference between the epistemological, logical, and *ideal* definition of knowledge and its phenomenological experience. In few words, what feels like knowledge could be not so: what John Woods (2005) calls “epistemic bubble” defines the easily experienced condition in which we realize that we cannot distinguish, from our first-person perspective, what we know and what we just believe we know. Of course, this condition that feels like knowledge can also involve a form of deliberate not-knowing. However, the mere presence of parts of knowledge or ignorance should imply that we used irrational reasoning to get to that state. As Jefferson et al. (2017, p. 7) point out: “Epistemically irrational beliefs and predictions can be either true or false, but what makes them irrational is that they were not formed on the basis of (sufficiently robust) evidence or are insufficiently responsive to evidence after being adopted.”

So, of course, the epistemically problematic trait of irrational reasoning is not that they lead the agents to certain falsity, but that agents delude themselves thinking they have the appropriate epistemic resources to make a decision when it is not the case. Here we will specifically comment on three patterns of reasoning that are considered irrational by most authors in philosophical and psychological literature: self-deception, epistemic akrasia, and wishful thinking. We selected these types of irrational reasoning and not others (as superstition or prejudice) because they all involve types of deliberate not-knowing at their core. So, to discuss the role of deliberate not-knowing in these phenomena, it would not be enough to establish that agents end up being more ignorant than expected in the end, but how and why deliberate not-knowing shapes these kinds of reasoning. To reflect upon these issues, we will briefly present the main definitions of these psychological phenomena, and we will then dedicate a part of the explanation to the comment on the role of ignorance in their maintenance and its motivational character.

### 2.1. Wishful Thinking

Wishful thinking is commonly described as a positive illusion (Jefferson et al., 2017) which generally moves the agents to believe in statements corresponding to their wishes and to avoid believing ones that are inconsistent with their motivations (Sigall et al., 2000; Mayraz, 2011). This general description does not firmly separate wishful thinking from other kinds of biased reasoning, such as the ones tainted by confirmation bias, and tends to see the motivational character of human reasoning in a theoretical competition with epistemic reasons. Of course, according to the theoretical purposes of different authors, the definition of wishful thinking can become more specific or more general. Some use “wishful thinking” to describe any situation in which “hopes, fears, needs, and other motivational factors combine with, or compete with, prior beliefs as people confront scientific evidence and discourse” (Bastardi et al., 2011) or to refer to how people “avoid information or resist revising their beliefs […] in the competition between cognition and motivation” (Kruglanski et al., 2020).

So it is easy to judge wishful thinking as irrational because, in most cases, the epistemic reasons fall back in the competition with non-epistemic reasons (hope, fear, needs), and the reasoners unjustifiably consider their beliefs epistemically sound. Of course, as Kruglanski et al. (2020) specify, the fact that wishful thinking may reasonably be considered irrational does not mean that it is uncommon in our ordinary decision-making process. On the contrary, we often experience the competition between what we should believe and what we hope/want/need to believe, and we do not always consciously make the epistemically sound “choice” between them.

Thus, in the case of wishful thinking, deliberate not-knowing appears in two ways: a selection of information that discards what goes against the agents' interests and a general unawareness regarding the non-epistemic ground for the doxastic outcomes. So, we claim that willful ignorance, as the “general avoidance of situations that let someone aware of certain information, evidence, or knowledge” could better describe the kind of deliberate not-knowing enacted in these cases. While wishful thinking, people need to avoid certain information and preserve the “wishful” attitude—which consists of a more or less blissful unawareness regarding the effect of non-epistemic reasons on their judgment. Instead, people who avoid particular knowledge are well aware of which information they are avoiding and why, so they are, by definition, not wishfully thinking.

### 2.2. Epistemic Akrasia

The case of epistemic akrasia is complicated since it involves a contrast between first-degree and second-degree orders of beliefs. The general definition says that “epistemic akrasia is possible only if (a) a person's (first-order) beliefs diverge from his higher-order judgments about what it would be reasonable for him to believe and (b) these divergent (first-order) beliefs are freely and deliberately formed” (Owens, 2002, p. 19). In other words, epistemic akrasia describes the situation in which agents hold a belief even though they think it is irrational or unjustified (Greco, 2014; Daoust, 2019; Coates, 2020).

The reasons why they hold this belief is what identifies the akratic pattern of reasoning from other irrational ones: pragmatical akrasia—or weakness of will—is the situation in which people have all qualities, motives, and opportunity to do something that they think would be right for them and fail to do so because they lack conviction, will, and so they give in to the temptation to do easier but less good actions. In a similar way, when people give in and adopt beliefs for epistemic akrasia, they do not want to perform those analytic and epistemically righteous judgments that would allow them to reject some beliefs because of a lack of proof or the presence of counter-proofs to their evidence. They hold on to ignorance as they form false beliefs or insufficiently motivated ones because it is convenient in some respects.

If people choose not to “think hard enough” about what they believe, we can say that they may fall easily into a state of willful ignorance since this condition is broad and general enough to describe deliberate dismissal of adequate reasoning. At the same time, it would be unfair to claim that also knowledge avoidance has a role in this process. People who adopt epistemically akratic reasoning to form beliefs do not exactly know which kind of information, evidence, and knowledge they are dismissing, because they do not put enough effort into knowing that. The akratic reasoning prevents them from precisely selecting which information they are dismissing, so they are not definitely in a condition of knowledge avoidance.

### 2.3. Self-Deception

Finally, self-deception could represent a challenge to the idea that knowledge avoidance is less involved in irrational beliefs and patterns of reasoning than willful ignorance. The main reason is that we currently do not have only one definition of the phenomenon but multiple descriptions, upon which scholars are still debating. Indeed, Deweese-Boyd (2021) in the Stanford Encyclopedia of Philosophy presents the issue as such: “Virtually every aspect of self-deception, including its definition and paradigmatic cases, is a matter of controversy among philosophers […] self-deception involves a person who seems to acquire and maintain some false belief in the teeth of evidence to the contrary as a consequence of some motivation, and who may display behavior suggesting some awareness of the truth. Beyond this, philosophers divide […]” and begins a long list of issues that pertain to this topic.

We need to say, though, that even if it is fascinating to ponder the controversial issues surrounding self-deception, most of its problematic traits do not matter in this particular discussion—as, for example, its morality or practical efficacy. Instead, one controversial but relevant issue at hand is its *intentional* character^6^. Adopting accounts that differ on this particular matter can dramatically change its definition. According to Pedrini (2012) self-deception could have three distinct definitions relative to its intentional character:

1. people hold false beliefs while simultaneously knowing that they are false. They hold dear these false beliefs because it would be too painful to accept that they are false. This is usually called the *intentionalist account* (Davidson, 2004);
2. people delude themselves and believe something false because they have a desire that trumps epistemic reasons to believe otherwise. In this case, they do not know that they hold a false belief, but a thematic desire compromises their rational processes. This is usually presented as the *anti-intentionalist account of self-deception* (Mele, 2000);
3. people shift between believing a certain painful proposition to be true and a condition of self-delusion, in which they believe that proposition is false—*weak intentionalist account* (Pedrini, 2018);

In all three definitions, deliberate not-knowing is involved since agents believe in false statements for different reasons. The first definition is the easiest to dismiss as a case of knowledge avoidance. If self-deceiving people at the same time believe/suspect *p* (or have enough reasons/evidence to believe/suspect *p*) and refuse to acknowledge those beliefs and suspicions, they would no longer be in a position to avoid the information/knowledge that they wished they did not acquire. So, this condition would more easily encompass a state of willful ignorance, taken as a comprehensive phenomenon that includes the denial of evidence.

At this point, we need to point out that this definition of self-deception has been heavily criticized by the current philosophical literature, especially by Alfred Mele (2000), who talked about the paradox that surrounds it. Indeed, if we take “believing” as the condition that makes people say that something is true, then it is doubtful to assert that a person can believe at full force that something is both true and false. For this reason, Mele and other scholars have proposed the anti-intentionalist account of self-deception.

Mele and other anti-intentionalists (or non-intentionalists) (Johnston, 1995; Barnes, 2007), indeed, offer this description: subjects fall into self-deceiving patterns of reasoning when their epistemic motivations are compromised by the desire to believe a particular proposition. So, self-deceiving people would believe a particular false proposition *p* just because their *initial emotional motivation* to believe that *p* was more successful than epistemic motivations. Unfortunately, this poses another theoretical problem to the depiction of self-deception: within this theory, self-deception is only the *initial cause* for believing a false proposition, not the explanatory reason for its lasting effect. As Pedrini comments: “if a full-blown belief that *p* is successfully reached, then there is no trace of the psychological tension that seems, instead, to be highly typical of self-deception. For this tension is obviously due to the fact that the motivationally distorted self-deceptive process runs counter to evidence that not-*p* that is at hand, or that is easy available” (Pedrini, 2018, p. 2).

Within this account, we could not attribute the self-deceiving state to knowledge avoidance exactly because self-deceiving people do not recognize certain knowledge as available for emotional reasons (so they do not put any effort into avoiding certain information). In that sense, we are not even discussing a case of deliberate not-knowing since there is no non-epistemic motivation involved in the actual preservation of the state of ignorance.

On the contrary, weak intentionalist accounts of self-deception open the possibility that self-deceiving people would shift from a state of willful ignorance to knowledge avoidance and even self-delusion. Indeed, Pedrini argues that there is a tangible tension between believing and not believing a false proposition; it does not end up being a paradoxical situation, but the agents keep getting back and forth between believing the false proposition and recognizing it is false.

This definition of self-deception incorporates both kinds of deliberate not-knowing because when people are in a self-deluded state, they do not know they are ignorant even if this ignorance comes from their choices (willful ignorance). Instead, when they shift to a more self-knowing state, they still avoid gathering evidence in favor of the true proposition, so they forcefully maintain a condition of knowledge avoidance. Of course, neither willful ignorance nor knowledge avoidance depicts the complex process of self-deception entirely, even in this last and more complex characterization. Self-deception is the shifting between willful ignorance and knowledge avoidance, but neither of these conditions can comprehend the process of self-deception.

### 2.4. The Rationality of Knowledge Avoidance

As argued so far, commonly defined irrational phenomena mainly involve willful ignorance, not knowledge avoidance. Indeed, returning to the definition we offered of knowledge avoidance, we said that it describes a condition in which agents avoid some knowledge to refrain from anticipated costs (in terms of pain, anxiety, or regret) of possessing it. So knowledge avoidance does not technically involve the willful preservation of false beliefs or the generic dismissal of evidence in favor of certain theoretical positions. It instead refers to situations in which agents have not (nor look for) evidence to fixate a particular belief regarding a specific situation. If these cases do not fit the range of the irrational reasoning we so far described, how should we judge them? In these situations, people avoid acquiring those pieces of information that would impact their emotional state, reasoning abilities, and decisions. Is this another form of epistemic irrationality, or are they adopting patterns of reasoning closer to rational ignorance?

To proceed with our argument, we should point out that, so far, the examination of these cases adopted an old-fashioned cognitivist take on the matter. In many papers regarding this topic, the authors account for material costs of acquiring specific knowledge (money, time, etc.) but not the emotional response of the agents—so, nonmaterial costs. In this paper, we aim at partially closing this gap in the literature, discussing the impact of emotions and, in particular, epistemic feelings—which are feelings that depend on epistemic states (Arango-Muñoz, 2014a,b)—have on the human reasoning.

## 3. Epistemic Feelings, Anticipated Regret, and the Appeal to Autonomy

In the last two decades, philosophers and cognitive scientists have adopted some distinguishing features to discriminate between types of feelings, separating, for example, between emotional feelings and epistemic ones (Arango-Muñoz, 2014a). In particular, in studies regarding metacognition, feelings have been described as experiences that regard objects or states of affairs that affect the subjects' organism in certain specific ways. While emotional feelings are pretty known and fit without issues in this description, epistemic feelings are less understood and need more explanations to be comprehended in this framework. Epistemic feelings are phenomenal experiences regarding agents' cognitive abilities, conditions, or processes. So, while there is a bodily reaction that accompanies these experiences—to make a practical example, we can think of how it feels to have something on the tip of our tongue (tip-on-the-tongue feeling)—the trigger of these experiences is internal (Arango-Muñoz and Michaelian, 2014). Moreover, since epistemic feelings are reactions to internal contents, epistemic and emotional feelings can create loops between each other and chains of reaction. These reactions and loops, of course, happen without the explicit acknowledged approval of the subjects; instead, they profoundly affect them and their rational evaluations.

We here argue that we should consider the importance of epistemic feelings when reflecting upon knowledge avoidance. Indeed, from a theoretical point of view, it would be reasonable to admit that we can describe the anticipated regret of a decision as an epistemic feeling. The feeling of anticipated regret—which is the leading cause of knowledge avoidance according to Gigerenzer and Garcia-Retamero (2017)—rests upon the idea that we could not cope or we would not be happy with acquiring a particular knowledge (either because it would cause us too much pain because it would spoil our surprise, or it would make us unfair judges). So, at this point, we should discuss whether anticipated regret allows agents to perform types of rational reasoning or not.

In the next section, we will discuss possible conditions that may elicit anticipated regret. We will defend the idea that the anticipated regret of “no longer being as autonomous as before” may be considered a rational reason to avoid specific knowledge in the new bounded and ecological rationality standards. In particular, we will argue that when knowledge avoidance is conscious, and the agent is aware of the information is giving up (and of the feelings that this knowledge would trigger), we should see knowledge avoidance as evidence of embodied bounded rationality.

### 3.1. The Hoping Stand: Overcoming the Paradox of Autonomy

As already mentioned, (Gigerenzer and Garcia-Retamero, 2017) propose to take into account “anticipated regret” as one of the negative feelings that may arise when considering not acquiring a particular piece of information. We agree that we should consider anticipated regret as one reason for which people avoid acquiring specific knowledge. However, we think that there is more to add to this consideration: what could more accurately describe how anticipated regret works—considering it as an epistemic feeling—in the mind of people who avoid knowing certain things is the specification of its content. So, what is this anticipated regret about, and why should this potential content matter for the agent?

Here we propose to consider as an answer to this question the agent's *anticipated regret of no longer being as autonomous as before knowing certain information*. To defend this claim, we first need to specify what we can describe as “autonomy,” what we will name “epistemic autonomy,” and what one of us (Magnani, 2020) has named “the paradox of autonomy”.

So, autonomy is not an uncontroversial topic in philosophy, especially in ethical discussions. Buss and Westlung (2018) offer three different accounts of personal autonomy that they claim are dominant and interacting in the current philosophical literature. These accounts are labeled “coherentists” since they variously affirm that 1) agents are autonomous if they are motivated to act, and this motivation is coherent with some of their mental states (Frankfurt, 1971); 2) agents are autonomous when their actions are coherent with a “sufficiently wide range of reasons” for and against that behavior (these reasons could be based on facts about their desires and interests, or even false beliefs) that the agents know and can express (Fischer and Ravizza, 1998); then 3) “the essence of self-government is the capacity to evaluate one's motives on the basis of whatever else one believes and desires, and to adjust these motives in response to one's evaluations” (Buss and Westlung, 2018). We take the last account to describe an “epistemic” type of autonomy, to differentiate it from practical forms of it (those that have to do with “what agents can do” instead of “what agents can believe”).

Considering this last definition, the “paradox of autonomy” takes shape (Magnani, 2020). It claims that if, on the one hand, agents need reasoning to be autonomous—so they rely on their decisions, rules, preferences, and desires, on the other hand, the same decisions, rules, preferences, and desires can oppress our thinking and reduce our epistemic autonomy. Moreover, since we know that even our autonomous reasoning may lead to a reduction or an enhancement of our practical and epistemic autonomy, we should judge the rationality of our judgments, decision-making processes, and reasoning on how much the consequences of our decisions will allow us to preserve enough epistemic autonomy to make other rational choices.

With these critical points at hand, we need to reconsider the rationality of knowledge avoidance. Indeed, considering what we have described so far, we can provide some reasons to justify knowledge avoidance rationally. The anticipated cost of acquiring specific knowledge could affect the agent's epistemic autonomy and the agent's autonomy in general.

To explain the first reason adequately, we need to get back to discuss the intersections between cognition and emotions. Indeed, there is quite an emerging literature that describes negative emotions as more impactful on the cognitive capacity of agents than positive ones. Indeed, this realization brings out what Eil and Rao (2011, p. 116) call the “good news, bad news” effect:

Our primary finding is that subjects incorporated favorable news into their existing beliefs in a fundamentally different manner than unfavorable news. In response to favorable news, subjects tended to respect signal strength and adhered quite closely to the Bayesian benchmark, albeit with an optimistic bias. In contrast, subjects discounted or ignored signal strength in processing unfavorable news leading to noisy posterior beliefs that were nearly uncorrelated with Bayesian inference. […] We call this finding the good news bad news effect. The result suggests that bad news has an inherent “sting” that differential processing mitigates.

So, if agents anticipate that, by seeking out specific knowledge, they could receive news so bad that they would compromise their rational decision-making processes, it would be more reasonable to remain ignorant or postpone the acquisition of that knowledge to preserve solid reasoning-making abilities. Since the reasoning capacity is one of the conditions for maintaining both epistemic and practical autonomy, we can also justify this choice to defend one's autonomy in general.

Moreover, it is essential to consider also the degree of certainty that certain information carries. Let us consider two cases: 1) Amanda does not know if she has a genetic marker that would increase her possibility of suffering from a debilitating disease; 2) Beatrice is suffering from a disease now, but she has not received the diagnosis yet. If Amanda gets tested and receives a positive result, she will not be sure that she will suffer from that disease in the future. In the worst-case scenario, by being tested she would only know of a potential restriction of her future autonomy. In that case, she may choose to believe as if that restriction was a certainty, restricting her epistemic and practical autonomy even if she may never suffer from that particular disease. In this case, avoiding that knowledge would be an empowering choice for Amanda, which would increase her perceived epistemic and practical autonomy.

Beatrice, instead, is already in a condition that restricts her autonomy: receiving a diagnosis would allow her to take control and “ownership of her destiny” (Magnani, 2020). Choosing to not know, in her case, would amount to willful ignorance since she would not only need to avoid finding one information attainable by a medical test, but she would also need to avoid acknowledging any symptoms of her disease, recurring to wishful thinking, self-deception, and other irrational patterns of reasoning.

At the same time, it seems understandable that people would avoid knowledge regarding future states of affairs, especially if they believe they have control over their developments. For example, in a series of studies, Gigerenzer and Garcia-Retamero (2017) asked if people would like to know, with certainty, if their marriage would last or not. Most people refused this possibility. While the authors claim that the “anticipated regret” was at the heart of this decision, we claim that “the loss of perceived autonomy” could very well be the content of that particular epistemic feeling. Indeed, if there were the possibility of foreseeing how long a marriage would last, then it would mean that people do not have any power to change the situation. They would believe that they are not in charge of their relationship. So, choosing not to know seems the only way to preserve their epistemic autonomy, if not autonomy in general. We can put forward almost the same affirmation, even considering the case in which the test would predict with high accuracy (not certainty) the result. How would people know if knowing the result of the test will not affect the duration of their marriage? Knowing would imply a gamble in epistemic autonomy: if knowing the result of the test would affect the perception of people's autonomy, then it is reasonable to stay in a state of not-knowledge and preserve the perception of full epistemic and practical autonomy on the length of their marriage.

So, by not knowing, people are able to overcome the paradox of autonomy: by avoiding or postponing the acquisition of specific knowledge, they can preserve the perception of their epistemic and practical autonomy, and they would not have reasons to doubt that it is genuine. This refusal of “specific certainty or semi-certainty” is also confirmed by studies from (Kruglanski et al., 2020, p. 416):

Often, individuals crave specific certainty concerning beliefs they find reassuring, flattering, or otherwise pleasing. A student may prefer to know that they passed an exam, a patient may prefer to receive a clean bill of health, a suitor may prefer to have their affections returned. Similarly, one may avoid specific certainties that are troubling or threatening. Not knowing that one failed an exam is more pleasant than knowing that one did. Agnosticism concerning the alleged misconduct of one's child is preferable to unpleasant certainty in this matter. Avoidance of specific uncertainty can lead people to value ignorance.

Moreover, together with a curated perception of their autonomy, by not knowing certain information, people would also preserve a certain optimism that their future choices will be free and rational. So, in these cases, knowledge avoidance of certain data is less a preservation of a “blissful ignorance” and more a form of curation of a pragmatically valuable emotion: *hope*.

As Bloeser and Stahl (2017, p. 11) affirms, “hope is implicit in most pragmatic philosophies,” since it has not only to do with agents' expectations and desires, but also with the possibility that certain things will happen and on the actions that agents need to perform to make sure their hopes are not in vain. We need to add that hope is necessary to preserve the perception of both practical and epistemic autonomy. Indeed, it does not only preserves the idea that the future may reserve positive events but also that people can reason, form beliefs, and justify their actions to make them happen. Thus, if it preserves the agent from emotional costs or loss of autonomy, the choice of not-knowing will also preserve hopeful considerations on the future, which, in turn, will help agents to reason and form beliefs toward the further preservation of their autonomy.

### 3.2. The Appeal to Autonomy: Comparing Cases

As a last consideration, we need to compare how the appeal to autonomy can help us make a case for the rationality of knowledge avoidance but not willful ignorance. Let us review two cases^7^:

- Clara is a lawyer. She thinks she can better defend her client if she believes her client is innocent. Defending her client to the best of her ability is what she wants to do. She acquires evidence that her client is guilty, and she engages in self-deception—she starts looking for reasons to reject the evidence, however strong. She wants to maintain her belief in her client's innocence as then she would be better able to act as she wishes.
- Denise is also a lawyer, with the same ambition of defending her client and the same belief that she will do a better job if she believes the client is innocent. She has the chance to read a potentially incriminating letter. To maintain her epistemic autonomy, she refuses to read it.

Clara engages in self-deception as the intentionalists describe it and so falls into willful ignorance in order to—allegedly—better serve her client. Instead, Denise seems to choose the less committing option of knowledge avoidance: without acquiring the potential evidence of her client's guilt, she allows herself to free her judgment of the idea that her client might be guilty. Even if the situations seem similar, we still maintain that, while Clara is limiting herself by falling into a self-deceiving state, Denise may still have a chance to increase her epistemic autonomy.

Clara, in fact, both *knows* that her client is guilty and is in a state of denial regarding this fact. At the same time, she is not preventing others from finding evidence for her client's guilt because she is fooling herself regarding the client's innocence. So, instead of considering, for example, extenuating circumstances for her client's actions—which would increase her ability to have a fair trial for her client and ultimately serve better the client's interests—she is just burying her head in the sand. So, she is not increasing in any way her pragmatic or epistemic autonomy: she is just trapping herself in a self-deceiving pattern of reasoning, limiting her options to defend her client better.

Instead, Denise is in a precarious situation: by not reading the potentially incriminating letter, she has avoided acquiring the belief that the client might be guilty—or not. So, if that letter is the only potential evidence of her client's guilt and only she could present the evidence in court, by avoiding reading the letter, she is precluding herself to either acquire further evidence of her client's innocence or to have the chance to get rid of the only evidence that may prove her client's guilt. If the letter does not contain evidence of guilt, Denise is just preserving a belief that the letter would confirm–that her client is innocent. If the letter contains evidence of her client's guilt, by not reading the letter, she is not putting herself in a position of choosing between defending her client as guilty or defending her client as innocent and destroying the evidence. If Denise is aware that she would not easily make this choice or she would choose to destroy the evidence, potentially ruining her career if caught, she is preserving both her epistemic and pragmatical autonomy by not reading the letter.

Now, suppose that Denise's client is guilty, and that letter is *not* the only potential evidence of her client's guilt: other people could present evidence of her client's guilt in court. In that case, she will need to face another choice: falling into self-deception as Clara did or still defending her client by looking for extenuating circumstances for the client's actions.

So, by considering all the options the two lawyers face, we can still defend the idea that people who avoid specific knowledge, if they are aware of the emotional toll the acquisition of that knowledge would take on them, do increase their epistemic autonomy, while willfully ignorant people do not.

## 4. Conclusions

In this paper, we offered some reasons to defend the rationality of knowledge avoidance. To fully explain this epistemic right to not-know, we have first distinguished between “willful ignorance” and “knowledge avoidance”: while the former amounts to all cases in which people try to preserve a general state of ignorance (as doubt, uncertainty, indecision, etc.) also avoiding all circumstances that would allow them to stumble on particular knowledge by accident, the latter describes the agents' avoidance of a particular piece of information, which could not fall in their laps otherwise.

To defend the rationality of knowledge avoidance, we used takes from embodied cognition research and theories of bounded/ecological rationality (Goldstein and Gigerenzer, 2002; Bissoto, 2007; Spellman and Schnall, 2009; Xu et al., 2020). Even if the rationality-irrationality spectrum recently became more nuanced with the contribution of these theories, we reflected on the fact that there are still states and processes deemed irrational in the current literature. So, we asked ourselves which kind of deliberate not-knowing had a role in irrational patterns of reasoning, such as wishful thinking, self-deception, and akrasia, and we argued that, while willful ignorance has a significant role to play in these states, knowledge avoidance does not play a crucial part in most of them.

Then, we focused on the reasons for which knowledge avoidance could be considered rational. To proceed with our argumentation, we discussed the impact of certain feelings—epistemic ones—on people's reasoning abilities. Following the basic tenets of embodied cognition, we argued that the emotional impact of certain information should be considered among the costs of acquiring knowledge, contributing to judging certain situations of knowledge avoidance as rational. Moreover, we discussed the impact of knowledge avoidance on the agents' sense of autonomy, which also brought us to discuss the concepts of epistemic autonomy and the paradox of autonomy.

In sum, we maintained that if knowledge avoidance is fully conscious and agents are aware both of the information they are giving up and of the emotional impact that information would have if acquired, then rejecting to seek that knowledge is a form of rational and autonomy-increasing *hope-depended selection of information*. We, of course, do not claim that the appeal to autonomy is the only argument we can advance to defend the epistemic rationality of knowledge avoidance^8^. Nevertheless, we believe it is one reason to consider knowledge avoidance rational in a perspective of embodied bounded rationality.

## Author Contributions

SA wrote the first draft of the manuscript. Both authors contributed to manuscript revision, read, and approved the submitted version.

## Funding

Open access funding were provided by MIUR, Ministry of University and Research, Rome, Italy (grant no. PRIN 2017 Research 20173YP4N3) and by the University of Pavia (INROAd+ − ENIGMA).

## Conflict of Interest

The authors declare that the research was conducted in the absence of any commercial or financial relationships that could be construed as a potential conflict of interest.

## Publisher's Note

All claims expressed in this article are solely those of the authors and do not necessarily represent those of their affiliated organizations, or those of the publisher, the editors and the reviewers. Any product that may be evaluated in this article, or claim that may be made by its manufacturer, is not guaranteed or endorsed by the publisher.

## Footnotes

^1^We will define epistemic autonomy in section.

^2^Despite our intention to focus on whether we should consider willful ignorance and knowledge avoidance as part of *epistemically* irrational reasoning, we acknowledge that further considerations may be put out regarding how these conditions can be also part of irrational behaviors (deeming them as *pragmatically* irrational). However, discussing how knowledge avoidance and willful ignorance may be pragmatically rational or irrational is outwith the scope of this paper.

^3^“Tacit knowledge,” an epistemically positive term, may seem off place in a list of types of ignorance. On the contrary, the idea of tacit knowledge relies on the partial “unawareness” of the agent who is nonetheless competent. Polanyi's very motto “we can know more than we can tell” (Polanyi, 1966, p. 4) can be rephrase as “we can tell less than we can know,” and still it would describe a positive situation for the tacitly knowing agent. Indeed, in this paper, we will not use the word “ignorance” as loaded with a negative connotation, and we will comment on that point on the very beginning of section 2.

^4^Of course, since no scholar presented this distinction before, various authors tried to specify the phenomena they were interested in by coining other formulas—as “deliberate ignorance,” used by Gigerenzer and Garcia-Retamero (2017), to speak about what we are calling knowledge avoidance. Here we argue that, since *Ignorance Studies* now propose a more complex view of ignorance, “knowledge avoidance” should be preferred for accuracy in cases where people avoid knowing certain information.

^5^A clarification is needed at this point: in section 3 we will propose one pragmatic reason to consider knowledge avoidance epistemically rational. Even if it is a pragmatic reason to be in a specific epistemic state, we need to say that our argument will only be slightly connected to the debate on the pragmatic reasons for belief. Indeed, knowledge avoidance is not a way to form or maintain a particular belief but a way to avoid forming one. So, the pragmatic reason we will invoke supports the epistemic rationality of “suspending one's belief” instead of forming one.

^6^A terminological note here might be useful. In the philosophical debates on self-deception, the word “intentional” is only used to describe in which sense the agent who falls into a state of self-deception does it “deliberately.” Here we use the term with this meaning, and we will not refer to the notion of “intentionality” as “aboutness,” as it is commonly used in philosophy of mind.

^7^We need to thank one of the anonymous reviewers for challenging our theory by providing us these captivating scenarios.

^8^One of the anonymous reviewers pointed out, and we agree, that, for example, the account of the rationality of belief put forward by Fileva (2018) may justify the rationality of knowledge avoidance as well. On her view, the belief of the agent who engages in knowledge avoidance is consistent with the agent's evidence, so it does not violate evidential constraints, and so is at least minimally rational or rationally permissible even if it is not rationally ideal. Not so in the case of the willfully ignorant agent who forms beliefs contrary to the evidence.

## References

[B1] Arango-Muñoz S. (2014a). Metacognitive feelings, self-ascriptions and mental actions. Philos. Inquiries 2, 145–162. 10.4454/philinq.v2i1.81

[B2] Arango-Muñoz S. (2014b). The nature of epistemic feelings. Philos. Psychol. 27, 193–211. 10.1080/09515089.2012.732002

[B3] Arango-Muñoz S. Michaelian K. (2014). Epistemic feelings, epistemic emotions: review and introduction to the focus section. Philos. Inquiries 2, 97–122.

[B4] Arfini S. (2019). Ignorant Cognition. A Philosophical Investigation of the Cognitive Features of Not-Knowing. Cham: Springer.

[B5] Arfini S. (2021). Situated ignorance: the distribution and extension of ignorance in cognitive niches. Synthese 198, 4079–4095. 10.1007/s11229-019-02328-0

[B6] Arfini S. Magnani L. (2021). Embodied, Extended, Ignorant Minds. New Studies on the Nature of Not-Knowing. Cham: Synthese Library. Springer International Publishing.

[B7] Bardone E. (2011). Seeking Chances: From Biased Rationality to Distributed Cognition. Berlin; Heidelberg: Springer Science & Business Media.

[B8] Barnes A. (2007). Seeing Through Self-Deception. Cambridge: Cambridge University Press.

[B9] Bastardi A. Uhlmann E. L. Ross L. (2011). Wishful thinking: Belief, desire, and the motivated evaluation of scientific evidence. Psychol. Sci. 22, 731–732. 10.1177/0956797611406447 21515736

[B10] Bertolotti T. Arfini S. Magnani L. (2016). Abduction: From the ignorance problem to the ignorance virtue. J. Logics Appl. 3, 153–173.

[B11] Bissoto M. L. (2007). Self-organization, embodied cognition and the bounded rationality concept. Ciências Cognição 11, 80–90.

[B12] Bloeser C. Stahl T. (2017). Hope. Stanford: The Stanford Encyclopedia of Philosophy.

[B13] Bortolotti L. (2010). Delusions and Other Irrational Beliefs. Oxford: Oxford University Press.

[B14] Bortolotti L. (2014). Irrationality. Malden, MA: John Wiley & Sons.

[B15] Buss S. Westlung A. (2018). Personal Autonomy. Stanford: The Stanford Encyclopedia of Philosophy.

[B16] Chemero A. (2011). Radical Embodied Cognitive Science. Cambridge: MIT press.

[B17] Coates A. (2020). Rational epistemic akrasia. Am. Philos. Q. 49, 113–124.

[B18] Daoust M.-K. (2019). Epistemic akrasia and epistemic reasons. Episteme 16, 282–302. 10.1017/epi.2018.630886898

[B19] Davidson D. (2004). Problems of Rationality. Oxford: Oxford University Press.

[B20] Deweese-Boyd I. (2021). Self-Deception. Stanford: The Stanford Encyclopedia of Philosophy.

[B21] Eil D. Rao J. M. (2011). The good news-bad news effect: asymmetric processing of objective information about yourself. Am. Econ. J. Microecon. 3, 114–138. 10.1257/mic.3.2.114

[B22] Fileva I. (2018). What does belief have to do with truth? Philosophy 93, 557–570. 10.1017/S003181911800033530886898

[B23] Fischer J. M. Ravizza M. (1998). Responsibility and Control: A Theory of Moral Responsibility. Cambridge: Cambridge University Press.

[B24] Frankfurt H. G. (1971). Freedom of the will and the concept of a person. J. Philos. 68, 5–20. 10.2307/2024717

[B25] Gallagher S. (2018). Embodied rationality, in The Mystery of Rationality, eds Bronner G. Di Iorio F. (Cham: Springer International Publishing), 83–94.

[B26] Gettier E. L. (1963). Is justified true belief knowledge? Analysis 23, 121–123. 10.1093/analys/23.6.121

[B27] Gigerenzer G. Garcia-Retamero R. (2017). Cassandra's regret: the psychology of not wanting to know. Psychol. Rev. 124, 179–196. 10.1037/rev0000055 28221086

[B28] Gigerenzer G. Goldstein D. G. (1996). Reasoning the fast and frugal way: models of bounded rationality. Psychol. Rev. 103, 650–669. 10.1037/0033-295X.103.4.650 8888650

[B29] Goldstein D. G. Gigerenzer G. (2002). Models of ecological rationality: the recognition heuristic. Psychol. Rev. 109, 75–90. 10.1037/0033-295X.109.1.75 11863042

[B30] Greco D. (2014). A puzzle about epistemic akrasia. Philos. Stud. 167, 201–219. 10.1007/s11098-012-0085-3

[B31] Grossman Z. van der Weele J. J. (2017). Self-image and willful Ignorance in social decisions. J. Eur. Econ. Assoc. 15, 173–217. 10.1093/jeea/jvw001

[B32] Haack S. Kolenda K. (1977). Two fallibilists in search of the truth. Proc. Aristotelian Soc. Suppl. 51, 63–104. 10.1093/aristoteliansupp/51.1.63

[B33] Haas J. Vogt K. M. (2015). Ignorance and investigation, in Routledge International Handbook of Ignorance Studies, eds Grossand M. McGoey L. (Abingdon: Routledge), 17–25.

[B34] Jefferson A. Bortolotti L. Kuzmanovic B. (2017). What is unrealistic optimism? Conscious Cogn. 50, 3–11. 10.1016/j.concog.2016.10.005 27815016 PMC5380125

[B35] Johnston M. (1995). Self-deception and the nature of mind, in Philosophy of Psychology: Debates on Psychological Explanation, ed Macdonald C. (Cambridge: Blackwell), 63–91.

[B36] Jordan J. (1996). Pragmatic arguments and belief. Am. Philos. Q. 33, 409–420.

[B37] Kruglanski A. W. Jasko K. Friston K. (2020). All thinking is ‘wishful' thinking. Trends Cogn. Sci. 24, 413–424. 10.1016/j.tics.2020.03.004 32284177

[B38] Le Morvan P. (2013). Why the standard view of ignorance prevails. Philosophia 41, 239–256. 10.1007/s11406-013-9417-6

[B39] Le Morvan P. Peels R. (2016). The nature of ignorance: two views, in The Epistemic Dimensions of Ignorance, eds Peels R. Blaauw M. (Cambridge: Cambridge University Press), 12–32.

[B40] Mackie G. (2012). Rational ignorance and beyond, in Collective Wisdom: Principles and Mechanisms, eds Landemore H. Elster J. (Cambridge: Cambridge University Press), 290–318.

[B41] Magnani L. (2020). Autonomy and the ownership of our own destiny: tracking the external world and human behavior, and the paradox of autonomy. Philosophies 5, 12. 10.3390/philosophies5030012

[B42] Mastrogiorgio A. Petracca E. (2016). Embodying rationality, in Model-Based Reasoning in Science and Technology, eds Magnani L. Casadio C. (Cham: Springer International Publishing), 219–237.

[B43] May V. M. (2006). Trauma in paradise: willful and strategic ignorance in Cereus Blooms at Night. Hypatia 21, 107–135. 10.1111/j.1527-2001.2006.tb01116.x

[B44] Mayraz G. (2011). Wishful thinking. SSRN Electronic Journal, Paper n 1955644.

[B45] McIntyre L. (2015). Respecting Truth: Willful Ignorance in the Internet Age. London: Routledge.

[B46] Mele A. (2000). Self-Deception Unmasked. Princeton: Princeton University Press.

[B47] Oster E. Shoulson I. Dorsey E. R. (2013). Optimal expectations and limited medical testing: Evidence from huntington disease. Am. Econ. Rev. 103, 804–830. 10.1257/aer.103.2.804 29547253

[B48] Owens D. (2002). Epistemic akrasia. Monist 85, 381–397. 10.5840/monist200285316

[B49] Pedrini P. (2012). What does the self-deceiver want? Humana Mente 20, 141–157.34478746

[B50] Pedrini P. (2018). Liberalizing self-deception. les ateliers de l'Ethique. Ethics Forum 13, 11–24. 10.7202/1059496ar

[B51] Polanyi M. (1966). The Tacit Dimension. London: Routledge & Kegan Paul.

[B52] Reisner A. (2009). The possibility of pragmatic reasons for belief and the wrong kind of reasons problem. Philos. Stud. 145, 257–272. 10.1007/s11098-008-9222-4

[B53] Rubin D. I. (2018). Willful ignorance and the death knell of critical thought. New Educator. 14, 74–86. 10.1080/1547688X.2017.1401192

[B54] Sicherman N. Loewenstein G. Seppi D. J. Utkus S. P. (2016). Financial attention. Rev. Financ Stud. 4, 863–897. 10.1093/rfs/hhv073

[B55] Sigall H. Kruglanski A. Fyock J. (2000). Wishful thinking and procrastination. J. Soc. Behav. Pers. 15, 283–296.1469598

[B56] Simon H. A. (1997). Models of Bounded Rationality. Cambridge: MIT Press.

[B57] Somin I. (2015). Rational ignorance, in Routledge International Handbook of Ignorance Studies, eds Gross M. McGoey L. (Abingdon: Routledge), 274–281.

[B58] Spellman B. A. Schnall S. (2009). Embodied rationality. Virginia Public Law and Legal Theory Research, 35:Paper No. 17.

[B59] Star D. (ed.). (2018). The Oxford Handbook of Reasons and Normativity, 1st Edn. Oxford, United Kingdom; New York, NY: Oxford University Press.

[B60] Sweeny K. Melnyk D. Miller W. Shepperd J. A. (2010). Information avoidance: Who, what, when, and why. Rev. General Psychol. 14, 340–353. 10.1037/a0021288

[B61] Todd P. M. Gigerenzer G. (2007). Environments that make us smart: ecological rationality. Curr. Dir. Psychol. Sci. 16, 167–171. 10.1111/j.1467-8721.2007.00497.x

[B62] Todd P. M. Gigerenzer G. E. (2012). Ecological Rationality: Intelligence in the World. Oxford: Oxford University Press.

[B63] Werner K. (2021). Cognitive confinement: theoretical considerations on the construction of a cognitive niche, and on how it can go wrong. Synthese 198, 6297–6328. 10.1007/s11229-019-02464-7

[B64] Williams D. (2021). Motivated ignorance, rationality, and democratic politics. Synthese 198, 7807–7827. 10.1007/s11229-020-02549-8

[B65] Woods J. (2005). Epistemic bubbles, in We Will Show Them! Essays in Honour of Dov Gabbay, Vol. 2, eds Artemov S. Barringer H. d'Avila Garcez A. Lamb L. C. Woods J. (London: College Publications), 731–774.

[B66] Xu F. Xiang P. Huang L. (2020). Bridging ecological rationality, embodied emotion, and neuroeconomics: Insights from the somatic marker hypothesis. Front. Psychol. 11:1028. 10.3389/fpsyg.2020.01028 32581926 PMC7286429

[B67] Zimmerman M. J. (2018). Recklessness, willful Ignorance, and exculpation. Crim. Law Philos. 12, 327–339. 10.1007/s11572-017-9424-y

